# Defining Microbiota‐Derived Metabolite Butyrate as a Senomorphic: Therapeutic Potential in the Age‐Related T Cell Senescence

**DOI:** 10.1111/acel.70257

**Published:** 2025-11-07

**Authors:** Nia Paddison Rees, Jessica Conway, Ben Dugan, Sayeda S. Amir, Aimee Parker, Simon R. Carding, Niharika A. Duggal

**Affiliations:** ^1^ Department of Inflammation and Aging, School of Infection, Inflammation and Immunology University of Birmingham Birmingham UK; ^2^ Quadram Institute, Norwich Research Park Norwich UK; ^3^ National Institute for Health and Care Research (NIHR) Birmingham Biomedical Research Centre Birmingham UK

**Keywords:** ageing, cellular senescence, inflammation, T cell

## Abstract

Advancing age is accompanied by an accumulation of senescent T cells that secrete pro‐inflammatory senescence‐associated secretory phenotype (SASP) molecules. Gut‐microbiota‐derived signals are increasingly recognised as immunomodulators. In the current study, we demonstrated that ageing and the accumulation of senescent T cells are accompanied by a reduction in microbial‐derived short‐chain fatty acids (SCFAs). Culturing aged T cells in the presence of butyrate suppresses the induction of a senescence phenotype and inhibits the secretion of pro‐inflammatory SASP factors, such as IL6 and IL8. Administration of faecal supernatants from young mice rich in butyrate prevented in vivo accumulation of senescent spleen cells in aged mice. The molecular pathways governing butyrate's senomorphic potential include a reduced expression of DNA damage markers, lower mitochondrial ROS accumulation, and downregulation of mTOR activation, which negatively regulates the transcription factor NFκB. Our findings establish butyrate as a potent senomorphic agent and provide the evidence base for future microbiome restitution intervention trials using butyrate supplements for combating T cell senescence, ultimately reducing inflammation and combating age‐related pathologies to extend lifelong health.

## Graphical abstract

An age‐associated accumulation of senescent T cells is characterized by increased cellular damage and the secretion of pro‐inflammatory molecules collectively known as the senescence‐associated secretory phenotype (SASP). In the present study, we demonstrate that this accumulation of senescent T cells in aged hosts is accompanied by a reduction in microbial‐derived short‐chain fatty acids (SCFAs). Notably, culturing aged T cells in the presence of butyrate suppresses the induction of the senescence phenotype and inhibits the secretion of pro‐inflammatory SASP factors, such as IL‐6. Mechanistically, the senomorphic potential of butyrate is linked to reduced expression of DNA damage markers, decreased mitochondrial ROS accumulation, and downregulation of mTOR signalling, which in turn negatively regulates the transcription factor NFκB. Together, these findings identify butyrate as a potent senomorphic agent and provide a rationale for future microbiome‐restoration trials using butyrate supplementation to mitigate T cell senescence, reduce inflammation, and ultimately combat age‐related pathologies to promote healthy longevity.

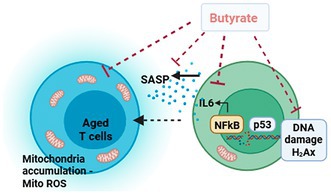

## 1 Introduction

Advancing age is accompanied by remodelling of the immune system and an impaired ability to mount robust immune responses, termed immunosenescence (Duggal 2018). Replicative stress is associated with repeated antigenic stimulation across the life course, induces a state of cellular senescence, in aged T cells. This is characterised by cell cycle arrest in response to damage dominantly in memory T cells, which is mediated by tumour suppressor p53 in response to persistent DNA damage (Akbar et al. 2016). An increased senescent T cell burden is a hallmark of immunosenescence, and these cells have been reported to be enriched in the CD45RA^+ve^CCR7^−ve^ EMRA antigen experienced memory T cell population that expands with age. Despite displaying a state of cell cycle arrest in response to stressors, such as DNA damage, senescent T cells are still functional and possess a unique secretome of a range of pro‐inflammatory cytokines, such as IL6, IL8 and TNFα, which are collectively termed senescence‐associated secretory phenotype (SASP) (Callender et al. 2018). Importantly, a recent study in mice revealed that driving premature T cell senescence using genetic strategies induces multiple ageing‐related features, including musculoskeletal, cardiovascular and cognitive alterations, highlighting an important role of senescent T cells in accelerating ageing (Desdín‐Micó et al. 2020). Moreover, senescent T cells can contribute to development of a persistent state of basal inflammation in aged participants termed inflammaging (Dugan et al. 2023) and reduce the ability of older adults to combat infections as confirmed by the higher mortality rate in older adults from SARS‐Cov2 infection (Covre et al. 2020). An accumulation of senescent T cells has also been reported in age‐related diseases, including rheumatoid arthritis (Raza et al. 2025), Alzheimer's disease (Gate et al. 2020) and cardiovascular diseases (Youn et al. 2019), suggesting that clearance of these cells could have geroprotective benefits. A frequent approach to eliminate senescent cells from aged tissues and organs is treatment with senolytic drugs or curtailing/neutralising the production of SASP components using senomorphics, such as metformin and rapamycin (Tchkonia et al. 2013). Despite showing promise senomorphics possess broad immunosuppressive effects (Baroja‐Mazo et al. 2016), limiting their widespread adoption and highlighting the need to identify of safer senomorphic therapies for promoting healthy ageing.

The human gastrointestinal tract (GIT) houses a complex ecosystem of microbes, the microbiome, comprising diverse microbial communities and products of their metabolism that contribute to maintaining health and promoting the development and functioning of the immune system (Levy et al. 2017). Among the resident bacteria, *Bacteriodetes* and *Bifidobacteria* ferment non‐digestible dietary fibres to produce short‐chain fatty acids (SCFAs), that function as key regulators of host health by promoting intestinal epithelial barrier integrity and serving as a main energy source for colonocytes (Rivière et al. 2016). Butyrate possesses immunomodulatory functions, such as promoting the expansion of *T*_regs_ (Furusawa et al. 2013) and *B*_regs_ (Rosser et al. 2020), stimulating anti‐inflammatory processes associated with protection from auto‐immune mechanisms. Furthermore, butyrate can promote the memory potential and antiviral cytotoxic effector functions of CD8 T cells (Bachem et al. 2019), and B cell differentiation and secretion of IgA and IgG antibodies (Kim et al. 2016). However, to date no studies have systematically investigated how butyrate influences the function of aged immune cells.

GIT physiology and its microbiome composition changes with age, characterised by a reduction in SCFA‐producing microbes (e.g., *Faecalibacterium prausnitzii* , *Roseburia species*) and the expansion of opportunistic pathogens (pathobionts), leading to intestinal microbial dysbiosis (Buford 2017). This produces changes in microbial metabolite profiles and decreased levels of faecal SCFAs, such as butyrate and propionate, but also other microbial‐derived metabolites, including secondary bile acids, with advancing age; all of which play a crucial role in maintaining intestinal barrier integrity and immune homeostasis (Salazar et al. 2019). The impact of ageing on the GIT microbiome and its associations with immune senescence and inflammaging is an area of active research (Conway and Duggal 2021). Consistent with this predicted association, germ‐free mice are protected from microbiome dysbiosis and intestinal barrier dysfunction and do not display features of inflammaging or macrophage ageing (Thevaranjan et al. 2017). Further evidence of this causal relationship comes from a human faecal microbiota transplant (FMT) study in which transplanting faecal samples from a young donor into aged *C. difficile* infected patients increased faecal levels of SCFAs, particularly butyrate, in association with restoring microbiome homeostasis and producing a decrease in peripheral senescent T cell numbers (Monaghan et al. 2021).

In this study, we sought to better understand the host‐microbiome‐metabolome‐immune axis by investigating the role of butyrate in modulating aged T cell function such as cytokine release (SASP factors) in a subset of senescent‐like T cells using an in vitro assay. Considering that butyrate production declines with age, if the potential benefits of this metabolite in governing the activities of senescent cells are proven, butyrate supplementation will be a valuable novel therapeutic target in future geriatric medicine for maintaining immune homeostasis throughout the ageing process.

## 2 Materials and Methods

### 2.1 Study Design, Blood and Stool Sample Collection

Heparinized peripheral blood and paired stool samples were collected from 40 healthy young (aged 18–37 years; 17 males) and 40 healthy aged donors (aged ≥ 60 years; 18 males; 8 of these aged donors were invited for a follow‐up visit for in vitro assays) from whom informed consent was obtained for a study approved by the HRA (Health Research Authority) and Health and Care Research Wales (HRCW) Approval (IRAS 301974). All participants had no infection at the time of testing, no known immunodeficiency, inflammatory diseases, or chronic diseases, or any history of chemotherapy or radiotherapy and were not receiving any immunosuppressive medications within the last 6 months. Each donor provided an 18 mL blood sample alongside a paired faecal sample.

### 2.2 LC–MS Analysis

To 1 g of faeces, 5–10 ceramic microbeads were added, and the vial was shaken at 500×*g* for 10 min at 20^ο^C. Samples were then centrifuged at 4800×*g* for 10 min at 20^ο^C and the collected supernatant was filtered by passing through a Whatman Uniprep filter vial (0.45 μm). 100 μL of the cleaned extract was taken into a glass autosampler vial. Derivatisation to the nitrophenylhydrazine was made by the addition of 50 μL each of 50 mM 3‐Nitrophenylhydrazine hydrochloride (3NPH) in 80% methanol, 50 mM dimethylaminopropyl‐N‐ethylcarbodiimide hydrochloride (EDC) in 80% methanol, and 7% pyridine in methanol. The mixture was shaken and heated at 40°C for 60 min before terminating the reaction by the addition (250 μL) of 0.5% aqueous formic acid. The sample was then passed for analysis by LC‐MS/MS. Calibration standards were prepared by using plasma purchased from Sigma as a blank matrix and fortifying with SCFAs at 100–12 μg/mL. This enabled matrix‐matched standards for direct comparison of detected response. All standards were then prepared in the same manner as the samples. Samples were analysed by LC‐MS using a Waters Acquity UPLC coupled to a Waters TQS‐μ mass spectrometer operated in positive multiple reaction mode (MRM). The analytical column was a Waters HSS T3 C18 100 × 2.1 mm × 1.7 μ at 35°C. Mobile phases were solvent A: water with 0.1% formic acid and solvent B: acetonitrile with 0.1% formic acid with a constant flow of 0.5 mL/min. A gradient was applied as 10% B at injection for 1 min, 23% B at 4.5 min, 50% B at 6 min, 100% B at 7 min, and held for 1 min, then back to initial conditions for 3 min. Sample injections were 2 μL. Filtrates were quantified to a limit of quantification of 25 μg/mL in the final solution alongside butyric acid standards (50–1000 μg/mL). For quantification, D2‐propionic acid was used as an internal standard for butyrate. The mass spectrometer was operated in positive electrospray selected ion monitoring mode for parent ion (89.12), fragment ion (43), cone voltage (20), and collision energy (10).

### 2.3 Replicative Senescence Assay in T Cells

Peripheral blood mononuclear cells (PBMCs) were isolated utilising Ficoll‐Paque density gradient centrifugation, and T cells were purified by negative selection (Easysep) according to the manufacturer's instructions. Freshly isolated T cells (1 × 10^6^ cells/mL) were incubated with 2 μg/mL of purified anti‐CD3 mAb (Thermo Fisher, UK) in 96 well‐round bottom microtitre plates at 37°C for 72 h in the presence of butyrate (1 mM; Sigma Aldrich). Conditioned medium was collected by centrifugation at 700 × g for 3 min at 20^ο^C and stored at −80°C until further analysis. IL6, IL8 (CXCL8), IL1β, and CCL3 (MIP1α) concentrations were measured in diluted supernatants (1:50 dilution for IL6 and IL1β ELISAs, 1:100 dilution for CCL3 ELISA, 1:200 dilution for IL8 ELISA) using ELISA duo kits (R&D Systems) as per manufacturer's instructions.

### 2.4 T Cell Phenotyping

Post culture, washed cells were stained with a combination of antibodies: 2 μg/mL CD3 PEcy7 (clone UCHT1; eBioscience, Invitrogen, Hatfield, UK), 5 μg/mL CD4 eFluor 450 (clone OKT4; eBioscience), 3 μg/mL CD45RA PerCP/cy5.5 (clone HI100; Biolegend), 8 μg/mL CCR7 APCcy7 (clone G043H7; Biolegend), 6 μg/mL CD57‐FITC (clone TB01; ebiosciences), and 0.5 μg/mL CD28‐APC (clone CD28.2; eBioscience) for 20 min on ice in the dark. For intracellular staining, the cells were fixed for 15 min, followed by permeabilisation using the transcription factor staining buffer set (eBioscience) and stained with the following antibodies: 5 μg/mL p‐p53 APC (clone: 184727; R and D Systems), 5 μg/mL γ H2AX PE (clone: CR55T33; Thermo Fischer), 5 μg/mL NFkB PE (clone: B33B4WP; eBiosciences), and 5 μg/mL Ki67 FITC (clone: SolA15; eBiosciences) for 20 min at room temperature. Apoptosis was assessed using Annexin V staining. All samples were run using a Miltenyi MACS Quant (BD Milteny Biotech, Germany) flow cytometer and analysed using FlowJo v10.8.1 software (BD Life Sciences, UK). Concentration‐matched isotype controls set (eBioscience) were used to set the gates, and single‐fluorochrome stained controls were used to compensate for spectral overlap. Flow cytometry data were analysed using Flow Jo v10.8 software (BD Biosciences, USA).

### 2.5 Detecting Phosphorylated p38 and p‐S6 by Flow Cytometry

p38 (pT180/pY182) and p‐S6 (mTORC1 target) expression by PBMCs after co‐culture (30 min) with Ionomycin (500 ng/mL; Sigma Aldrich, UK) and phorbol myristate acetate (PMA) (50 ng/mL; Sigma Aldrich, UK) at 37°C in the presence or absence of butyrate. Cells were then washed and stained with a combination of anti‐human CD3, CD4, CD45RA, and CCR7 cell surface antibodies for 20 min in the dark on ice. Cells were then washed and fixed in 4% paraformaldehyde (PFA) for 30 min at room temperature in the dark. Post incubation, 1 mL of ice‐cold methanol (100%) was added, and the cells were incubated for 10 min on ice. Subsequently, cells were incubated for 30 min in the dark at room temperature with anti‐human 5 μg/mL p38 PE (BD Biosciences) or anti‐human 5 μg/mL p‐S6 PE (BD Biosciences). Post incubation, cells were washed, and samples were acquired using a Miltenyi MACS Quant (BD Miltenyi Biotech, Germany) flow cytometer and analysed using FlowJo v10.8.1 software (BD Life Sciences, UK) to determine frequencies of positive cells and expression levels (MFI) within.

### 2.6 Autophagic Flux (LC3‐II)

Measurement of autophagic flux in T cells was performed using a flow cytometry antibody‐based LC3 assay kit (Luminex) as per the manufacturer's instructions. T cells post culture with or without butyrate were incubated with or without Reagent A for 2 h. Cells were harvested post wash in PBS for 15 min at 4^ο^C, followed by cell surface staining with anti‐human CD3, CD4, CD45RA, and CCR7 cell surface antibodies for 20 min in the dark on ice. Post incubation, cells were washed in 1× assay buffer diluted in dH_2_O. Cells were then permeabilised using 1× Reagent B for 5 min in the dark on ice and centrifuged at 700×*g* for 5 min. Pelleted cells were then resuspended in 1:20 LC3‐II FITC monoclonal antibody diluted in 1× assay buffer and incubated for 30 min in the dark at 20^ο^C. Finally, T cells were washed in 1× assay buffer before finally being resuspended in PBS and analysed on the Miltenyi MACS Quant (BD Miltenyi Biotech, Germany) flow cytometer. Autophagic flux was calculated using the following formula: (LC3_Reagent A −_ LC3_Control_)/LC3_Control_.

### 2.7 Senescence‐Associated β‐Galactosidase SA‐βGal Staining

SA‐ßGal activity was detected using the Cellular Senescence Detection Kit (Dojindo Molecular Technologies), according to the manufacturer's instructions. Briefly, T cells (1 × 10^6^ cells/mL) were incubated with 1× bafilomycin A1 solution for 1 h at 37°C. Subsequently, cells were incubated with 1X SPiDER‐ßGal for 30 min at 37°C. Cells were centrifuged at 700×*g* for 5 min at 21°C and washed twice in PBS before finally being resuspended in PBS and analysed on the Miltenyi MACS Quant (BD Miltenyi Biotech, Germany) flow cytometer (Martinez‐Zamundio et al. 2021).

### 2.8 ImageStream Assessment of DNA Damage

Post‐culture, T cells were stained to assess DNA damage with phosphorylated histone γH_2_AX, and samples were acquired first by single‐colour fluorescence (to optimise the laser strength); then, experimental samples were run on the calibrated ImageStream IS100 (Amnis) for simultaneous acquisition of bright field, scatter, and 12 fluorescent images for each cell. The data files stored as rif files were analysed using IDEAS 4.0.735 software. Doublets and debris were excluded using the bright field area vs. aspect ratio feature and in‐focus single cells to locate the distribution of signals within cells. The intensity of γH_2_AX foci within the nuclear region identified by DAPI staining was quantified for the evaluation of DNA damage.

### 2.9 MitoTracker and MitoSOX Staining

Freshly isolated T cells were stained with either 100 nM MitoTracker green or 2 μM MitoSOX (Thermo Fisher) mitochondrial dyes for 30 min at 37°C. Cells were washed with 300 μL of non‐sterile PBS at 250×*g* for 5 min at 4°C and subsequently stained with a combination of anti‐human CD3, CD4, CD45RA, and CCR7 cell surface antibodies for 20 min in the dark on ice. Following this, cells were washed again in 300 μL of non‐sterile PBS at 250×*g* for 5 min at 4°C; the supernatants were discarded, and unfixed cells were resuspended in 300 μL of non‐sterile PBS for immediate flow cytometric and ImageStream analysis and recording of the MFI values within T cell subsets.

### 2.10 Gene Expression Analysis

Total RNA was isolated from 4 × 10^6^ T cells from untreated and butyrate‐treated T cells using the RNeasy Mini isolation kit (Qiagen, Germany). RNA concentrations and quality were measured using the Agilent 2100 BioAnalyzer. Gene expression analysis was performed using the Pan‐Cancer Immune Profiling Panel from NanoString Technologies (NanoString, USA), containing probes for 730 immune‐related genes and 40 housekeeping genes, representing 24 different immune cell types and common checkpoint inhibitors that cover adaptive and innate immune responses. For each sample, 80 ng of total RNA, with a maximum volume of 7 μL (> 28.6 ng/μL), was used. Hybridisation was performed at 65°C for 17 h using a SimpliAmp Thermal Cycler (Applied Biosystems, UK). The nCounter Flex system (NanoString, USA) was used for sample preparation. Raw gene counts were normalised using the most stable housekeeping genes from the panel. The background threshold was determined as the average count of the negative controls + 2 standard deviations. Differential expression of genes between control versus butyrate‐treated was tested with Mann–Whitney *U* tests, and Benjamin‐Hochberg procedures were used to correct for multiple testing.

### 2.11 Animal Experiments

All mouse experiments were carried out in compliance with the Home Office regulations under project licence P723e9201 and in accordance with FELASA guidelines and were approved by the University of East Anglia Animal Welfare and Ethics Regulatory Board (AWERB). Male pathogen‐free C57Bl6 mice (aged 24 months; *n* = 4) were housed in individually ventilated cages, fed a standard chow diet, provided with water ad libitum, and maintained under a 12‐h light: 12‐h dark cycle. Existing microbiota were depleted by delivery of a 3‐day broad‐spectrum antibiotic cocktail regime through combined oral gavage of unpalatable antibiotics (100 μL of vancomycin, 5 mg/mL; metronidazole, 10 mg/mL), while others were able to be provided in drinking water (ampicillin 1 g/L, neomycin 0.5 g/L). Post‐antibiotic washout, recipients were rehoused in clean cages and were orally administered 100 μL of a faecal water (20 mg stool from *n* = 10 young donors was pooled, vortexed, and resuspended in PBS, centrifuged to pellet bulk material, and 0.22 μm filtered) or 100 μL PBS, thrice weekly for 4 weeks. Spleens were collected 24 h after the final gavage and were frozen in OCT and stored at −80°C. Tissues were sectioned (7 μm) using a Leica CM1950 cryostat at −18°C to −20°C and fixed in 4% paraformaldehyde in PBS for 10 min TEMP, washed 2× for 5 min with 1× TBS, and permeabilized with 0.1% Triton X‐100 in PBS for 10 min TEMP. Sections were first incubated with 1% BSA at 20°C for 1 h, followed by anti‐rabbit p 53 (ab16048; 1:1000 dilution) for 16 h at 4°C. Sections were then washed 3× for 5 min with 1X‐TBS and then incubated with an anti‐rabbit IgG, Alexa Fluor 594 conjugated secondary polyclonal antibody (goat; A‐11037; 1:1000 dilution) for 1 h at 20°C. Sections were mounted with Prolong Diamond mounting media with DAPI. For immunofluorescence, images were acquired using a Zeiss Axio microscope. Images were composed and edited using ImageJ software (https://imagej.nih.gov/ij/), optimal brightness and contrast adjustments were applied uniformly across the entire image. Image analysis was quantified using the same software.

### 2.12 qRT PCR Analysis

RNA was isolated from 5 to 10 mg of spleen using the Qiagen lipid RNA isolation kit protocol. RNA quantity and quality were determined using a NanoDrop One Spectrophotometer and a 2100 Bioanalyser. Quantitative q RT PCR was carried out on 5 ng/μL RNA isolated from mouse spleen samples using the iTaq Universal SYBR Green One‐Step Kit (Biorad) on a CFX384 Tough Real‐Time PCR Detection System (Biorad). Primer sequences were p53 (forward primer TCCGAAGACTGGATGACTGC; reverse primer GATCGTCCATGCAGTGAGGT), p16 (forward CGAACTCTTTCGGTCGTACCC and reverse CGAATCTGCACCGTAGTTGAGC), and IL6 (forward primer CTGCAAGAGACTTCCATCCAG; reverse primer AGTGGTATAGACAGGTCTGTTGG). The PCR thermocycler condition was as follows: initial reverse transcription at 50°C for 10 min, polymerase activation at 95°C for 5 min, 40 cycles of denaturation at 95°C for 10 s, annealing at 60°C for 30 s, and initial elongation at 65°C for 31 s followed by 60 cycles of elongation at 65°C for 5 s. All samples were run in triplicate. Relative gene expression was calculated followed by normalisation of the values to the relative gene expression of Epcam.

### 2.13 Statistical Analysis

GraphPad Prism version 9 was used to perform all statistical analyses. Inter‐experiment comparisons in T cells cultured in the presence versus absence of butyrate were analyzed by paired test. The bar graph data represented mean ± SEM. Differences were considered significant when *p* was < 0.05.

## 3 Results

### 3.1 Age‐Associated Lowered Stool Microbial Metabolite SCFA Levels Correlate With Changes in CD28 ^−ve^ CD57 ^+ve^ Senescent T Cells

**FIGURE 1:**
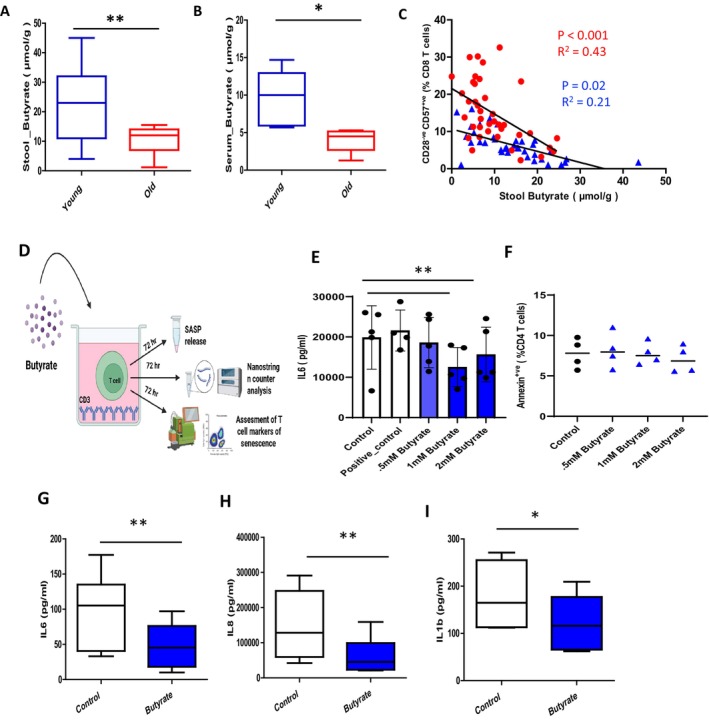
Stool butyrate levels correlate with peripheral accumulation of senescent T cells in older adults and characterisation of Senomorphic properties of butyrate and attenuation of SASP phenotype Box whisper plots showing (A) serum (B) stool butyrate levels in healthy young individuals (blue; *n* = 36) and healthy aged participants (*n* = 39) as measured by high‐performance liquid chromatography (C) Scatterplots show the correlation between stool butyrate levels and senescent CD8 T cell frequency in the peripheral blood of healthy young individuals (blue; *n* = 36) and healthy aged participants (*n* = 39). (D) Schematic of experimental design indicating culturing of aged T cells in CD3 coated plates with SCFA butyrate for 72 h (*n* = 6) and collection of cell‐culture supernatants for determining SASP features by ELISA and assessment of features of senescence via flow cytometry (E) CD3 treated T cells were incubated in the absence of butyrate (control), positive control (1 mM sodium bicarbonate) and with different concentrations of butyrate (0.5–2 mM) for 72 h. At the end of treatment, IL6 concentration in ell‐culture supernatant was measured by ELISA (*n* = 5) and (F) cell death was assessed via Annexin V positive staining. The secretion of (G) IL6 (H) IL8 (I) IL1β from CD3 treated aged T cells in the presence of 1 M butyrate. (J) IL6^+ve^ senescent CD57^+ve^ CD4 T cells CD3 treated aged T cells in the presence of 1 M butyrate. Data are mean ± SEM of six independent experiments. Statistical analysis was performed by two tailed paired student's *t*‐test *p < 0.05, **p < 0.01.

Advancing age is accompanied by an accumulation of senescent CD28^−ve^CD57^+ve^ T cells, displaying a state of replicative arrest (Callender et al. 2018). Based on initial evidence that GIT microbiota‐derived SCFAs are essential to maintaining immunological homeostasis (Conway and Duggal 2021). We hypothesised that age‐associated microbiome dysbiosis and the resulting loss of SCFAs contribute towards the development of a senescent T cell phenotype. To test our hypothesis, we collected paired stool and blood samples from healthy young and aged participants. We have observed a decline in stool SCFA levels of butyrate (*p* = 0.008) in older adults (Figure 1A). Importantly, we reported for the first time a decline in serum levels of butyrate with age (*p* = 0.04) (Figure 1B). To investigate if changes in SCFA levels are associated with the accumulation of senescent T cells, peripheral blood senescent T cell frequencies were enumerated in paired blood samples from healthy young and old participants. Peripheral senescent CD28^−ve^CD57^+ve^ CD4 T cell frequency was negatively associated with stool butyrate levels in older adults (*p* < 0.001, *R* ^2^ = 0.43) (Figure 1C).

### 3.2 Senomorphic Properties of Butyrate: Dampening of the Senescence‐Associated Secretory Phenotype in Aged T Cells

To test whether butyrate possesses senomorphic properties in humans an in vitro cellular model of T cell senescence by subjecting healthy T cells to a 3‐day T cell‐specific activation with anti‐CD3 and we confirmed that continuous proliferation can induce DNA double‐strand breaks (DSBs) indicated by the increased expression of a marker of DNA damage (γH_2_AX) (Figure S1A), activation of p‐p53 known to elicit cell cycle arrest and (Figure S1B) increased frequency of Saβ Gal expressing cells (Figure S1C) and fewer Ki‐67, a known marker of cell proliferation expressing cells (Figure S1D). Furthermore, upregulation of SASP component IL6 was confirmed in cell conditioned medium (Figure S1E) and intracellularly in senescent CD57^+ve^ T cells (Figure S1G).

This in vitro senescence assay was used to explore the ability of butyrate to modulate SASP features (senomorphic properties). The data obtained (Figure 1D) showed a significant decline in the levels of IL6 in conditioned media of aged T cell treated with 1M and 2M butyrate (Figure 1E). As shown by Annexin V staining this occurred in the absence of any change in cell viability (Figure 1F). Importantly, 1 mM butyrate promoted a significant reduction in the secretion of signature SASP features, including IL‐6, *p* = 0.003 (Figure 1G), IL‐8, *p* = 0.008 (Figure 1H), IL1β *p* = 0.04 (Figure 1I) by aged T cells. This downregulation of SASP feature, IL6 by butyrate was more significant in young T cells *p* < 0.0001 (Figure S1F).

### 3.3 Butyrate Regulates Senescence Phenotype of Aged T Cells

**FIGURE 2:**
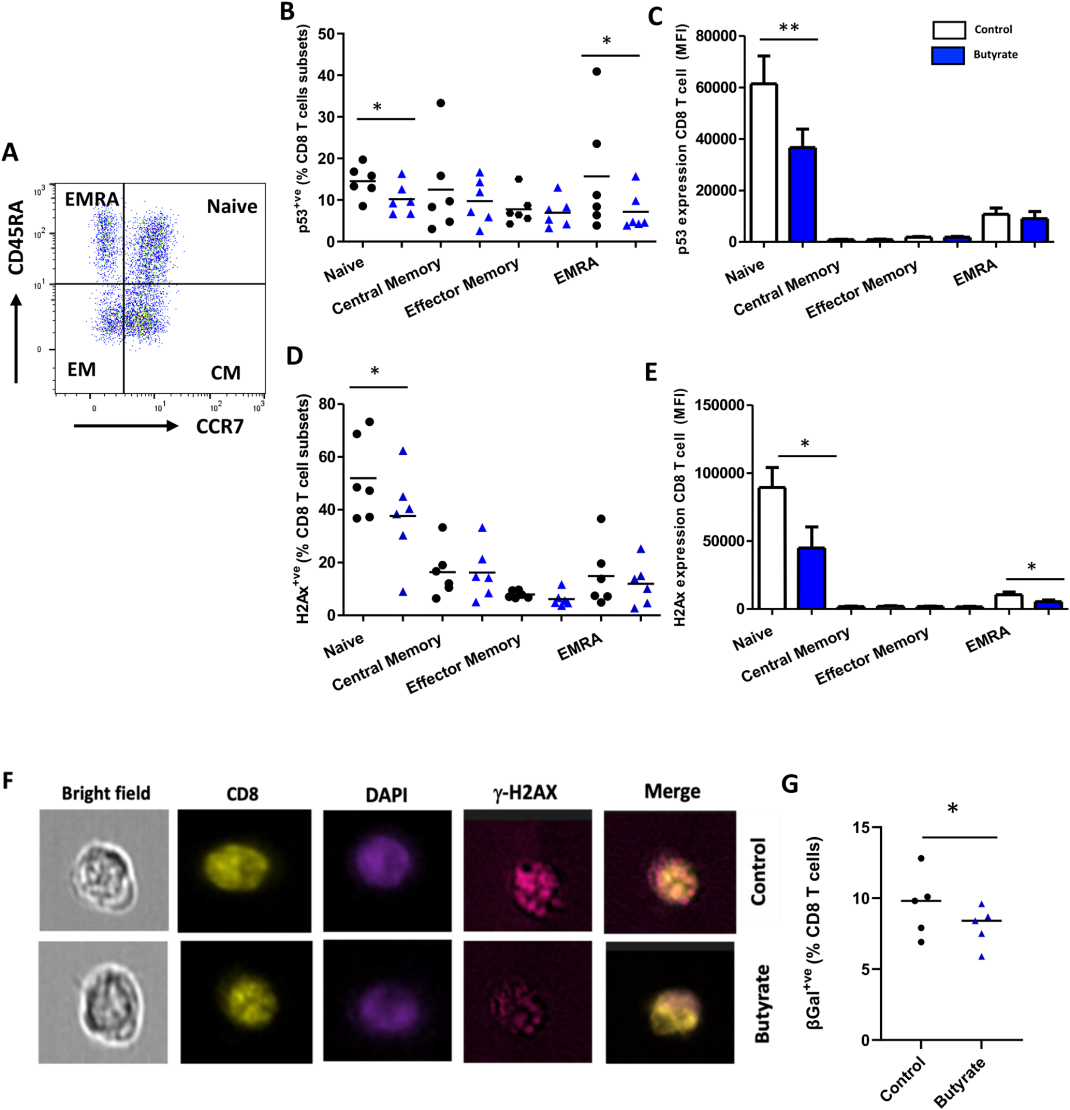
Regulation of senescence phenotype by butyrate in aged CD8 T cells (A) Representative flow cytometry showing gating strategy for T cell subsets based on phenotypic expression of CD45RA/CCR7 staining. In vitro culture of PBMCs in CD3‐coated wells for 3 days in the presence/absence of SCFA butyrate. On Day 3 (B) frequency of phosphorylated p53 expressing CD8 T cell subsets (C) phospho p53 expression levels in CD8 T cell subsets (D) frequency of γH2AX expressing CD8 T cell subsets (E) γH2AX expression levels (MFI) in CD8 T cell subsets. Statistical analysis was performed using a two‐tailed paired Student's *t*‐test. (F) Representative image stream images of cells stained with cell surface marker CD8, nuclear DAPI stain, and DNA damage marker γH2AX and merged image when PBMCs were cultured in CD3‐coated wells for 3 days in the presence/absence of SCFA butyrate. (G) frequency of SA‐βGal expressing T cell. The bar charts show expression data as the mean ± SEM of six experiments. *p = 0.05.

To determine whether butyrate impacts the exhibition of a senescent phenotype in T cell subsets, we compared the protein expression of phosphorylated p53 in naïve (CD45RA^+ve^CCR7^+ve^), central memory (CD45RA^−ve^CCR7^+ve^), effector memory (CD45RA^−ve^CCR7^−ve^), and terminally differentiated EMRA (CD45RA^+ve^CCR7^−ve^) CD4 and CD8 T cell subsets. Butyrate did not affect T cell subset distribution and frequency of Ki67‐expressing T cells (Table S1), a significant decrease in the frequency of phosphorylated p53‐expressing EMRA CD4 T cells (*p* = 0.02) (Figure S2A), naïve CD8 T cells (*p* = 0.01) and EMRA CD8 T cells (*p* = 0.03) (Figure 2A,B) was seen in butyrate‐cultured aged T cells compared to untreated controls. A similar decline in EMRA CD4 T cells (*p* = 0.02), and naïve CD8 T cells (*p* = 0.004) cultured in the presence of butyrate was also seen (Figure S2B, Figure 2C).

Next, we assessed if the SASP was dependent on the activation of ATM (Ataxia‐telangiectasia mutated) by measuring γH2AX, a direct target of ATM in response to DSBs. A significantly lower frequency of γH2AX‐expressing naïve CD4 T cells (*p* = 0.03; Figure S2C) and naïve CD8 T cells (*p* = 0.05; Figure 2D). Additionally, a decline in the expression (intensity) levels of γH2AX was observed in naïve CD8 T cells (*p* = 0.03) and EMRA CD8 T cells (*p* = 0.02) (Figure 2E). These findings were confirmed by the Image stream analysis (Figure 2F) showing reduced accumulation of DNA double‐strand breaks in butyrate‐treated cells.

### 3.4 Impact of Butyrate on Nuclear Factor Kappa B (NFκB), p38 MAPK, mTOR Signalling and Autophagic Pathways in Aged CD8 T Cells

**FIGURE 3:**
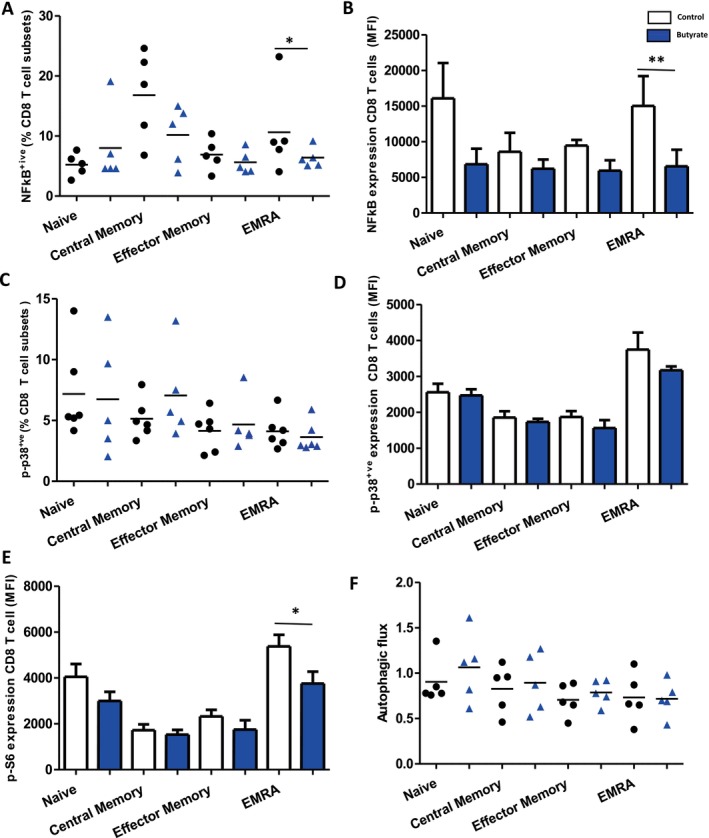
Effect of butyrate on nuclear factor kappa B (NFκB), p‐38 MAPK, mTOR signalling, and autophagic flux in aged CD8 T cells. In vitro culture of T cells in CD3‐coated wells for 3 days in the presence/absence of SCFA butyrate for (A) frequency of phosphorylated NF‐kB expressing CD8 T cell subsets, (B) NF‐kB expression levels in CD8 T cell subsets, (C) frequency of phosphorylated p38 expressing CD4 T cell subsets, (D) p‐p38 expression levels in CD4 T cell subsets, (E) pS6 mean fluorescence intensity (MFI), (F) autophagic flux in CD8 T cell subsets (naïve, central memory, effector memory, EMRA). A two‐tailed paired Student's *t*‐test performed the statistical analysis. Data are shown as the mean ± SD *p = 0.05, **p < 0.001.

To elucidate the mechanisms by which butyrate may influence the secretion of pro‐inflammatory cytokines by T cells, we investigated its impact on NFκB inhibition. A significant decrease in the frequency of phosphorylated NFκB‐expressing EM and EMRA CD4 T cells (*p* = 0.05 and *p* = 0.03, respectively) (Figure S3A) and EMRA CD8 T cells (*p* = 0.04) (Figure 3A) was seen in butyrate‐cultured T cells compared to untreated controls. A similar decline in EM and EMRA CD4 T cells (*p* = 0.01 and *p* = 0.02) and EMRA CD8 T cells (*p* = 0.02) was observed when cultured in the presence of butyrate (Figure 3B).

Examination of the frequency of phosphorylated p38‐expressing CD4 (Figure S3C) and CD8 T cells (Figure 3C) revealed no significant differences in butyrate‐cultured T cells compared to non‐treated controls. Similarly, p‐p38 expression levels showed no significant differences (Figure 3D, Figure S3D).

To test whether butyrate treatment blocked mTOR activity, we analysed ribosomal protein S6 kinase phosphorylation by flow cytometry. Butyrate treatment reduced expression of p‐S6 (mTORC1 target) in EMRA CD8 T cells (*p* = 0.03) (Figure 3E) but not CD4 T cell subsets compared to control T cells (Figure S3E).

To examine autophagy, we tested whether butyrate‐induced senomorphic properties were associated with increased autophagic processes. Butyrate treatment did not impact the autophagic flux in CD8 T cell subsets (Figure 3F) and CD4 T cell subsets (Figure S3F), based on comparing autophagy protein microtubule‐associated protein 1 light chain alpha (LC3) levels between cells treated with bafilomycin A or controls (Alsaleh et al. 2020).

### 3.5 Impact of Butyrate on Mitochondria Reactive Oxygen Species Generation

**FIGURE 4:**
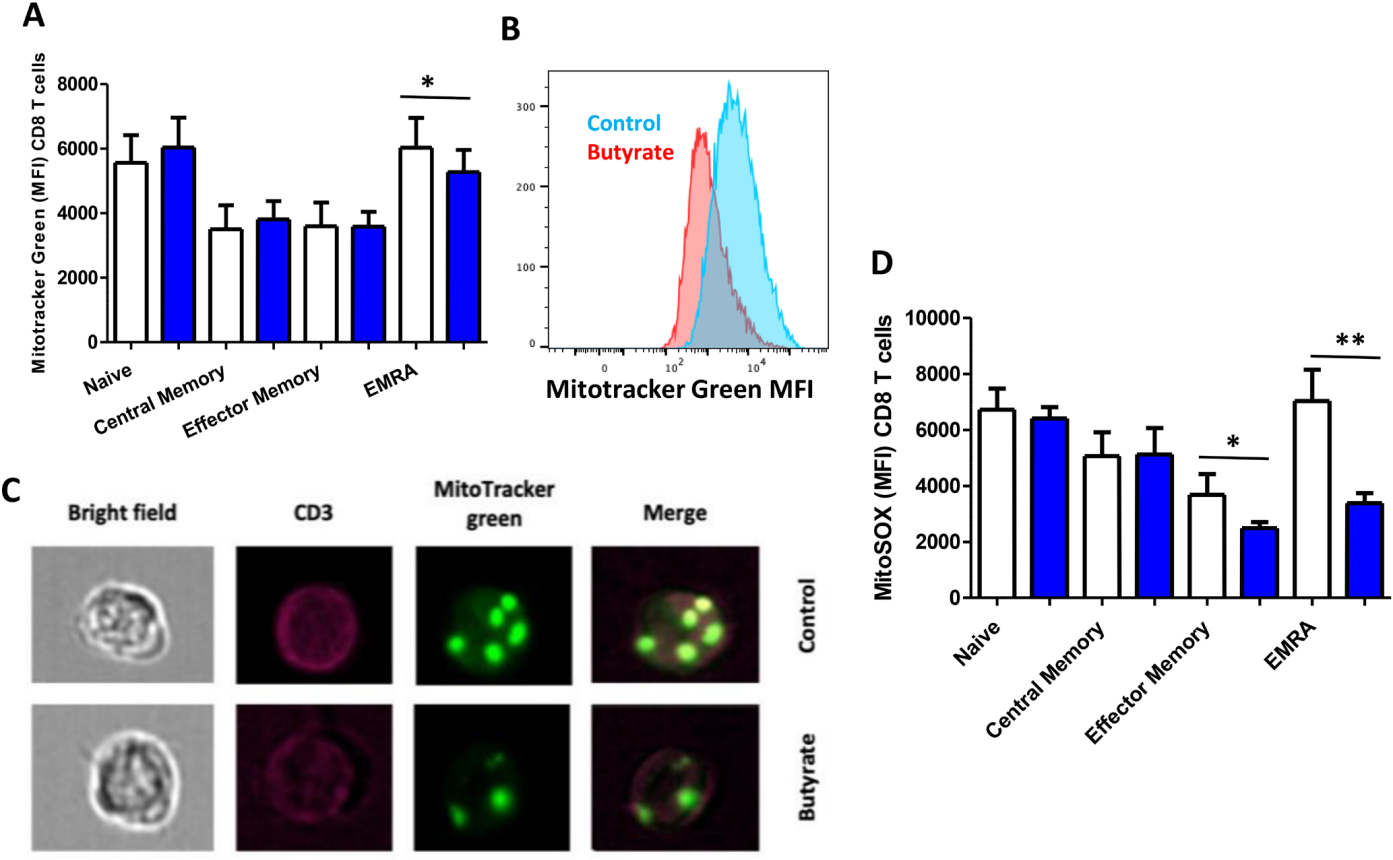
Effect of butyrate on mitochondrial mass and ROS production in CD8 T cells. In vitro culture of T cells in CD3‐coated wells for 3 days in the presence/absence of SCFA butyrate and stimulation. (A) MitoTracker green expression levels in aged CD8 T cell subsets (B) Representative histogram of the shift in MitoTracker green MFI between control (blue) and butyrate (red). (C) Representative ImageStream images of cells stained with cell surface marker CD3 and mitochondrial mass indicator MitoTracker green and subsequent merged images. (D) MitoSOX red expression levels in aged CD8 T cell subsets. Statistical analysis was performed by a two‐tailed paired Student's *t*‐test. Data are shown as the mean ± SD *p < 0.05, **p < 0.01.

We made use of the mitochondria‐specific MitoTracker green dye, which binds mitochondrial membranes in a mitochondrial membrane potential (MMP) independent manner, to determine changes in mitochondrial mass. A significant decline in MitoTracker green expression levels was seen in aged EMRA CD8 T cells (*p* = 0.02) cultured with butyrate (Figure 4A,B) and in aged EMRA CD4 T cells (Figure S4A). These results were confirmed via ImageStream analysis (Figure 4C). Damaged mitochondria are usually removed by the process of mitophagy (mitochondria‐specific autophagy), but this process too is downregulated in senescent cells, leading to a vicious cycle of further reactive oxygen species generation and DNA damage. ROS production measured using the MitoSOX Red mitochondrial superoxide indicator showed that there was a significant decrease in MitoSOX expression in aged EMRA CD8 T cells exposed to butyrate *p* = 0.001 (Figure 4D) but not in aged CD4 T cell subsets (Figure S4B).

### 3.6 Transcriptome Signature of Aged T Cells Post‐Butyrate Treatment

**FIGURE 5:**
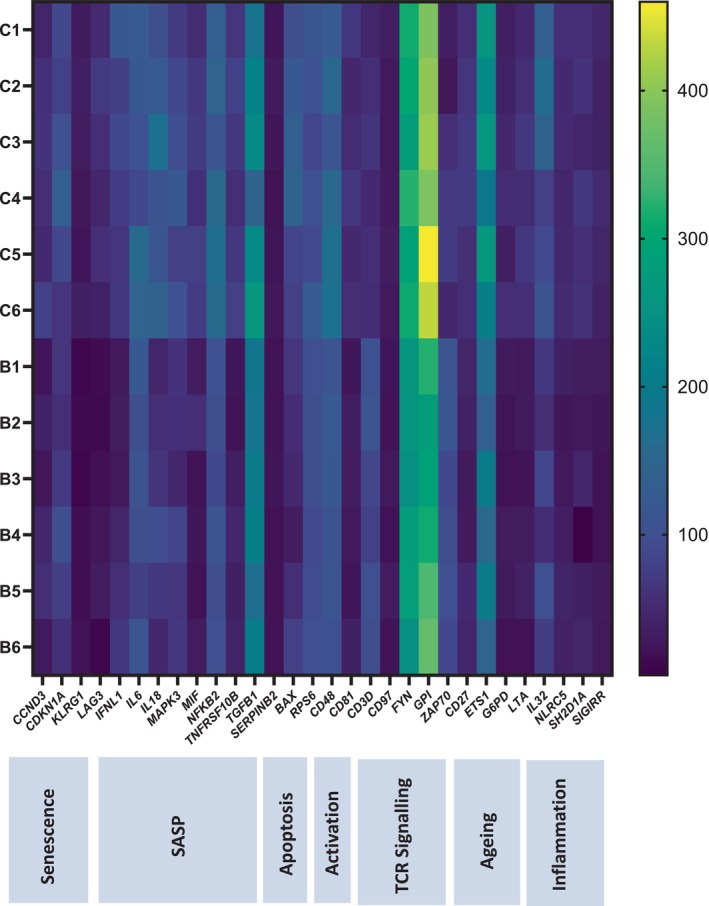
Transcriptome signature of aged T cells post butyrate treatment Heat map showing expression levels of differentially expressed genes in T cells isolated from six healthy aged participants cultured in CD3‐coated plates in the absence (control) or with 1 mM butyrate for 72 h.

To identify potential molecular signalling pathways in aged T cells that might contribute to the senomorphic properties of butyrate, we compared gene expression profiles using the Nanostring nCounter gene expression assay, allowing for the detection of 770 genes in T cells from six healthy aged donors that were cultured in the presence versus the absence of 1 mM butyrate for 72 h. First, we observed a translational repression in the mean gene expression levels of four senescence‐associated genes, such as cyclin‐dependent kinase inhibitors (CDKN1A), and nine SASP‐associated genes, such as IL6, SERPINB2, and others, that are part of the SenMayo gene set used to identify senescent cells (Saul et al. 2022) (Figure 5). An important limitation of our study is that cytokine secretion was measured in bulk T cell populations, making it difficult to distinguish general activation‐induced effects from changes specifically associated with a senescence‐associated secretory phenotype (SASP). Key signalling molecules regulating the SASP feature, such as NFκB, MAPK, MIF, and STAT3, that promote age‐associated changes in mitochondrial dynamics and T‐cell cytokine production (Zukowski et al. 2023), were downregulated post‐butyrate treatment. Senescent T cells have a distinct phenotype, including downregulated expression of the costimulatory molecules CD27 and high expression of killer cell lectin‐like receptor subfamily G member 1 (KLRG‐1) (Barbarin et al. 2017). In this study, we observed an upregulation of CD27 expression and downregulation of KLRG1 in butyrate‐treated aged T cells. Tumour suppressor protein, p53, is also known to upregulate anti‐apoptotic molecules, such as bax, which are often used as hallmarks of senescent cells. We observed that butyrate treatment downregulates the expression of bax, reducing their resistance to apoptosis. However, we observed an upregulation of key TCR signalling‐associated components (CD3, FYN, GPI, and Zap70) in butyrate‐treated aged T cells, which are known to be compromised in senescent T cells (Zhang et al. 2021). Lastly, we observed a downregulation in the expression of EST1, a known negative regulator of ribosomal biogenesis (Xiao et al. 2022), and pro‐inflammatory molecules, such as IL32. Together, our data suggest that butyrate has the capacity to restrain the expression of genes closely related to senescence and the SASP spectrum.

### 3.7 Treatment With Faecal Supernatants Rich in Butyrate Restricts Accumulation of Senescent T Lymphocytes in a Mouse Model

**FIGURE 6:**
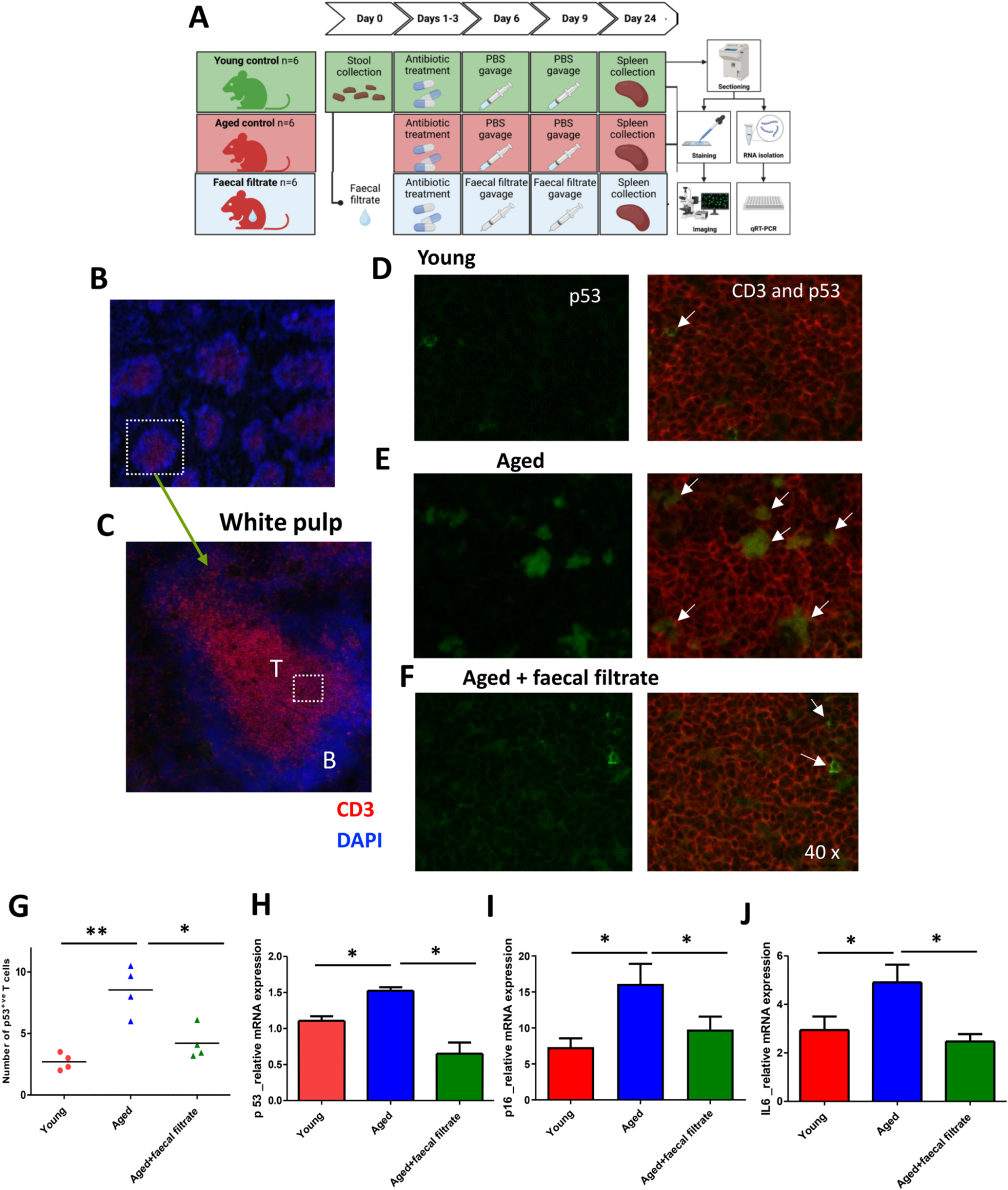
Influence of transfer of a young stool faecal filtrate to aged mice on the accumulation of senescent cells in the spleen (A) Overview of the experimental plan (B) Immunohistochemistry staining of the spleen with DAPI (nuclear stain) demonstrating white pulp regions (brighter blue regions) × 4 magnification (C) CD3 expressing T cell zone (red) × 10 magnification (D–F) merged images with arrows showing senescence marker p53 labelled with FITC (green) in CD3 expressing T cell zone × 40 magnification. (G) Graph representing the number of p53‐positive cells in the T cell zone of the spleen in 8 weeks young C57BL/6J mice (*n* = 4), aged C57BL/6J mice (*n* = 4) and aged mice receiving faecal filtrate from young mice = 4. Values are expressed as mean ± SEM. (H) RT‐q PCR‐mediated gene expression analysis of p53 (H) and p 16 (I) pro‐inflammatory cytokine IL6 (J) in the spleen of mice (*n* = 4 per group). *p = 0.05, **p < 0.001.

To validate the in vitro findings in an in vivo context, aged mice pre‐treated with a cocktail of antibiotics were gavaged with faecal filtrate from young mice that contain 0.3–1.2 μg/mg butyrate (Figure 6A,B). Immunohistology showed that the spleens of faecal filtrate‐treated aged mice had reduced numbers of senescent T cells (Figure 6C–F). CD3 and p53 dual staining revealed an increase in the number of senescent cells in aged wild‐type mice (*p* = 0.009) compared to young mice, whereas faecal filtrate treated aged mice had reduced levels of senescent p53^+^ T cells (*p* = 0.04) (Figure 6G). We also confirmed that the spleens of aged wild‐type mice exhibit increased p 53 mRNA and p 16 expression levels compared to young mice (*p* = 0.05 and *p* = 0.02, respectively), and that p53 mRNA and p 16 levels were significantly lower in faecal filtrate treated aged mice (*p* = 0.02, and *p* = 0.01 respectively) (Figure 6H,I). IL6 levels are correlates of inflammation, with the spleens of aged wild‐type mice exhibiting increased IL6 mRNA expression compared to young mice (*p* = 0.05). Consistent with faecal filtrates from young mice having a positive impact on inflammation, treated aged mice had significantly lower levels of IL6 mRNA compared with non‐treated aged animals (*p* = 0.04) (Figure 6J).

## 4 Discussion

Emerging evidence has highlighted the importance of a healthy gut microbiome in maintaining host health as we age by ameliorating the negative effects on immunosenescence and inflammaging (Conway and Duggal 2021). Therefore, interventions based on improving microbial homeostasis may be an effective strategy for improved senescence‐related phenotypes and extending healthy lifespan. While there is an abundance of preclinical evidence for the geroprotective effects of senolytics, the short‐term and long‐term safety profile and efficacy are yet to be fully investigated, strengthening the case for the identification of alternative senotherapeutics. Since many of the negative effects associated with senescence are driven by the SASP, senomorphics that modulate the SASP phenotype are emerging as a promising alternative therapeutic strategy. However, our understanding of the targeted effects of microbiome species and their metabolites in immune ageing remains limited. To our knowledge, the current study is the first to demonstrate that butyrate is a promising microbial metabolite possessing senomorphic properties at low millimolar dosages when cultured in vitro with aged T cells undergoing extensive proliferation through TCR activation. The effects of butyrate are mediated by downregulating mTOR activation, which targets transcriptional regulators of SASP, such as NFκB, and also ameliorates features of senescent T cells.

The proportion of T cells with a senescent phenotype increases with age but varies among individuals (Callender et al. 2018). We observed that an increased frequency of senescent‐like T cells was associated with reduced stool butyrate levels. Using an in vitro model of inducing senescence in T cells, we have demonstrated that butyrate prevents the acquisition of phenotypic characteristics of senescence and SASP features in activated primary T cells, confirming the senomorphic effects of butyrate. These findings are in line with a previous study reporting that circulating butyrate alleviates senescence in vascular cells of mouse aorta and reduces SASP expression (Han et al. 2018). Importantly, in vivo supplementation with stool supernatants from young stool rich in butyrate is accompanied by reduced accumulation of senescent cells in T cell zones of the spleen and inflammation in aged mice, providing scientific evidence that butyrate supplementation will be a valuable novel therapeutic target in future geriatric medicine for maintaining immune homeostasis through the ageing process.

Activation of stress response signalling cascades mediated via oxidative stress and DNA damage have been linked with activation of MAPKs, such as p‐p38, that govern the transcriptional programs in senescent cells and has been demonstrated to be a central driver of the SASP in senescent CD8 T cells and fibroblasts in vivo (Chambers et al. 2021; Sayegh et al. 2024). Mechanistically, our data suggest that in vitro butyrate treatment reduced the increased phosphorylation and nuclear accumulation of p‐p38. The p38 MAPK pathway is known to fuel DNA damage‐dependent activation of NFκB, triggering the production and secretion of SASP factors (Freund et al. 2011). We observed that butyrate suppresses NFκB activation, which is in line with another study reporting that butyrate inhibits NFκB activation in intestinal epithelial cells (Andoh et al. 1999) and human PBMCs (Usami et al. 2008).

mTOR activation contributes, at least in part, to driving a terminally differentiated T cell phenotype, as CD8 T cells from Tsc2^−/−^ mice experience T‐cell specific hyperactivity, display high positivity for T cell senescence, and exhibit proliferation dysfunction after antigenic challenge (Pollizzi et al. 2015). In our study, butyrate reduces S6 phosphorylation, a surrogate marker for inhibition of mTOR in treated aged T cells. Reductions in mTOR, which positively regulates glycolytic enzymes such as hexokinase II and glyceraldehyde 3‐phosphate dehydrogenase (Sun et al. 2011), may explain reduced levels of expression of this glycolytic enzyme seen here in butyrate treated T cells. Thus, the possibility that butyrate acts by inducing changes in metabolic activity in senescent cells that rely on glycolysis should be investigated further.

Dysfunctional mitochondria are a promoter of the secretory phenotype termed mitochondria dysfunction‐associated senescence (MiDAS) governed by a p53‐dependent pathway (Bektas et al. 2019). Additionally, mitochondria are the main producers of reactive oxygen species (ROS) that accelerate ageing by contributing to progressive cell damage and activation of stress pathways. Studies have reported that the elimination of mitochondria protects against pro‐inflammatory SASP features (Correia‐ Melo et al. 2016). Our data are consistent with butyrate mitigating mitochondrial ROS generation and reducing total mitochondrial mass build‐up in aged T cells (Callender et al. 2020) possibly by promoting the clearance of damaged mitochondria through mitophagy (selective degradation of dysfunctional and damaged mitochondria) or by inhibiting unnecessary mitochondrial biogenesis, which can be beneficial in enhancing mitochondrial quality.

A number of animal studies provide evidence of the anti‐inflammatory potential of butyrate and its use in treating lipopolysaccharide‐induced lung injury (Ni et al. 2010), preventing lethality of sepsis (Zhang et al. 2007), and atherosclerosis (Aguilar et al. 2014). SCFA are unsurprisingly being considered as supplementary treatment in the clinical management of inflammatory conditions. To date, two studies of butyrate administration have been reported in humans, one reporting successful amelioration of colonic inflammation in IBD after enema administration (Scheppach et al. 1992), and the other in older adults undergoing upper abdominal surgery observed an increase in butyrate concentration in portal vein blood (Van der Beek et al. 2015). These studies establish the safety of therapeutic administration of butyrate, although key questions regarding dose and formulation in older adults still remain unanswered, which requires further investigation before exploiting the potential of SCFAs as an anti‐immunosenescence intervention.

A key limitation of this study that needs to be acknowledged is that the 1 mM concentration may not accurately reflect physiological concentrations, which vary in vivo depending on tissue type and can be as high as 20–140 mM in the GI tract depending on intestinal microflora composition. We have observed serum levels as low as 0.1 mM, making it difficult to achieve the in vivo levels of butyrate in the T cell environment in an in vitro setting. Furthermore, a limitation of the mice experiments is that while the faecal supernatant from young mice was enriched in butyrate, it also contained a complex mixture of other microbial metabolites. Therefore, we cannot attribute the observed reductions in the build‐up of senescent cells in vivo solely to butyrate, and further studies using purified butyrate supplementation would be necessary to delineate its specific senomorphic properties in vivo.

In conclusion, our findings provide evidence in support of the use of butyrate to reduce the accumulation of senescent T cells with ageing by increasing butyrate levels through, for example, dietary, microbial, and therapeutic approaches. A nutritional approach to increase butyrate levels involves increasing the intake of high‐fibre foods, like fruits, vegetables, legumes, and whole grains, that serve as substrates for commensal microbes to produce SCFAs. Alternatively, the consumption of prebiotics, such as inulin and fructooligosaccharides, to selectively stimulate butyrate‐producing bacteria or the consumption of probiotics with butyrate‐producing bacteria are promising strategies. Further, our results provide the evidence base for a future clinical trial using oral sodium supplements or supplementation with butyrate precursors, such as tributyrin supplementation, to not only increase butyrate levels in aged hosts but to also rejuvenate aged T cells. However, this hypothesis needs to be strengthened, and there is room for future well‐planned studies in humans to help increase the translational potential of these findings.

## Author Contributions

Niharika A. Duggal gained funding for the study, participated in the design of the study, performed transcriptomic analysis, interpretation of the data, and wrote the first draft of the manuscript. Nia Paddison Rees participated in sample processing, performed the immune phenotyping, analyzed and interpreted the data generated. Jessica Conway recruited participants, participated in sample processing, performed the immune phenotyping, analyzed and interpreted the data generated for the first figure of the manuscript. Ben Dugan performed the ImageJ analysis. Simon R. Carding gained funding for the animal experiments and participated in its experimental design. Aimee Parker performed the animal experiments and collected the tissues for immunostainings. All authors edited and approved the final version of the manuscript.

## Conflicts of Interest

The authors declare no conflicts of interest.

## Supporting information


**Table S1:** T cell subset distribution post 3 day cell culture with Butyrate.
**Figure S1:** A cellular model of proliferation‐induced T cell senescence.
**Figure S2:** Regulation of senescence phenotype by butyrate in aged CD4 T cells.
**Figure S3:** Effect of butyrate on nuclear factor kappa B (NFκB), p‐38 MAPK and m TOR signalling in aged CD4 T cells.
**Figure S4:** Effect of butyrate on mitochondrial mass and ROS production in CD4 T cells.

## Data Availability

The raw flow cytometry data generated during the current study will be made available on Flow repository and the microscopy images generated during the current study will be shared via a google folder.

## References

[acel70257-bib-0045] Aguilar, E. C. , A. J. Leonel , A. R. Silva , et al. 2014. “Butyrate Impairs Atherogenesis by Reducing Plaque Inflammation and Vulnerability and Decreasing NFκB Activation.” Nutrition, Metabolism, and Cardiovascular Diseases 24: 606–613. 10.1016/j.numecd.2014.01.002. 24602606

[acel70257-bib-0001] Akbar, A. N. , S. M. Henson , and A. Lanna . 2016. “Senescence of T Lymphocytes: Implications for Enhancing Human Immunity.” Trends in Immunology 37, no. 12: 866–876. 10.1016/j.it.2016.09.002. 27720177

[acel70257-bib-0002] Alsaleh, G. , I. Panse , L. Swadling , et al. 2020. “Autophagy in T Cells From Aged Donors Is Maintained by Spermidine and Correlates With Function and Vaccine Responses.” eLife 9: 57950. 10.7554/elife.57950. PMC7744099 33317695

[acel70257-bib-0003] Andoh, A. , Y. Fujiyama , K. Hata , et al. 1999. “Counter‐Regulatory Effect of Sodium Butyrate on Tumour Necrosis Factor‐Alpha (TNF‐Alpha)‐Induced Complement C3 and Factor B Biosynthesis in Human Intestinal Epithelial Cells.” Clinical and Experimental Immunology 118: 23–29. 10.1046/j.1365-2249.1999.01038.x. 10540155 PMC1905403

[acel70257-bib-0004] Bachem, A. , C. Makhlouf , K. J. Binger , et al. 2019. “Microbiota‐Derived Short‐Chain Fatty Acids Promote the Memory Potential of Antigen‐Activated CD8^+^ T Cells.” Immunity 51: 285–297. 10.1016/j.immuni.2019.06.002. 31272808

[acel70257-bib-0005] Barbarin, A. , E. Cayssials , F. Jacomet , et al. 2017. “Phenotype of NK‐Like CD8^+^ T Cells With Innate Features in Humans and Their Relevance in Cancer Diseases.” Frontiers in Immunology 8, no. 316: 316. 10.3389/fimmu.2017.00316. 28396661 PMC5366313

[acel70257-bib-0006] Baroja‐Mazo, A. , B. Revilla‐Nuin , P. Ramirez , and J. A. Pons . 2016. “Immunosuppressive Potency of Mechanistic Target of Rapamycin Inhibitors in Solid‐Organ Transplantation.” World Journal of Transplantation 6: 183–192. 10.5500/wjt.v6.i1.183. 27011916 PMC4801794

[acel70257-bib-0007] Bektas, A. , S. H. Schurman , M. Gonzalez‐Friere , et al. 2019. “Age‐Associated Changes in Human CD4^+^ T Cells Point to Mitochondrial Dysfunction Consequent to Impaired Autophagy.” Aging 11: 9234–9263. 10.18632/aging.102438. 31707363 PMC6874450

[acel70257-bib-0008] Buford, T. W. 2017. “(Dis) Trust Your Gut: The Gut Microbiome in Age‐Related Inflammation, Health, and Disease.” Microbiome 5: 1–11. 10.1186/s40168-017-0296-0. 28709450 PMC5512975

[acel70257-bib-0010] Callender, L. A. , A. C. Caroll , E. A. Bober , et al. 2020. “Mitochondrial Mass Governs the Extent of Human T Cell Senescence.” Aging Cell 19: e13067. 10.1111/acel.13067. 31788930 PMC6996952

[acel70257-bib-0009] Callender, L. A. , A. C. Caroll , R. W. J. Beal , et al. 2018. “Human CD8^+^ EMRA T Cells Display a Senescence‐Associated Secretory Phenotype Regulated by p38 MAPK.” Ageing Cell 8: 12675. 10.1111/acel.12675. PMC5770853 29024417

[acel70257-bib-0011] Chambers, E. S. , M. Vukmanovic‐Stejic , B. B. Shih , et al. 2021. “Recruitment of Inflammatory Monocytes by Senescent Fibroblasts Inhibits Antigen‐Specific Tissue Immunity During Human Aging.” Nature Aging 1: 101–113. 10.1038/s43587-020-00010-6. 37118005

[acel70257-bib-0012] Conway, J. , and N. A. Duggal . 2021. “Ageing of the Gut Microbiome: Potential Influences on Immune Senescence and Inflammageing.” Ageing Research Reviews 68: 101323. 10.1016/j.arr.2021.101323. 33771720

[acel70257-bib-0013] Correia‐ Melo, C. , F. D. M. Marques , R. Anderson , et al. 2016. “Mitochondria Are Required for Pro‐Ageing Features of the Senescent Phenotype.” EMBO 35: 724–742. 10.15252/embj.201592862. PMC4818766 26848154

[acel70257-bib-0014] Covre, L. P. , R. P. H. De Maeyer , D. C. O. Gomes , and A. N. Akbar . 2020. “The Role of Senescent T Cells in Immunopathology.” Ageing Cell 12: e13272. 10.1111/acel.13272. PMC7744956 33166035

[acel70257-bib-0015] Desdín‐Micó, G. , G. Soto‐Heredero , J. F. Aranda , et al. 2020. “T Cells With Dysfunctional Mitochondria Induce Multimorbidity and Premature Senescence.” Science 368: 1371–1376. 10.1126/science.aax0860. 32439659 PMC7616968

[acel70257-bib-0016] Dugan, B. , J. Conway , and N. A. Duggal . 2023. “Inflammaging as a Target for Healthy Ageing.” Age and Ageing 52: afac328. 10.1093/ageing/afac328. 36735849

[acel70257-bib-0017] Duggal, N. A. 2018. “Reversing the Immune Ageing Clock: Lifestyle Modifications and Pharmacological Interventions.” Biogerontology 19: 481–496. 10.1007/s10522-018-9771-7. 30269199 PMC6223743

[acel70257-bib-0018] Freund, A. , C. K. Patil , and J. Campisi . 2011. “p38 MAPK Is a Novel DNA Damage Response‐Independent Regulator of the Senescence‐Associated Secretory Phenotype.” EMBO Journal 30: 1536–1548. 10.1038/emboj.2011.69. 21399611 PMC3102277

[acel70257-bib-0019] Furusawa, Y. , Y. Obata , S. Fukuda , et al. 2013. “Commensal Microbe‐Derived Butyrate Induces the Differentiation of Colonic Regulatory T Cells.” Nature 504: 446–450. 10.1038/nature12721. 24226770

[acel70257-bib-0020] Gate, D. , N. Saligrama , O. Leventhal , et al. 2020. “Clonally Expanded CD8 T Cells Patrol the Cerebrospinal Fluid in Alzheimer's Disease.” Nature 577: 399–404. 10.1038/s41586-019-1895-7. 31915375 PMC7445078

[acel70257-bib-0021] Han, Y. M. , T. Bedarida , Y. Ding , et al. 2018. “β‐Hydroxybutyrate Prevents Vascular Senescence Through hnRNP A1‐Mediated Upregulation of Oct 4.” Molecular Cell 71: 1064–1078. 10.1016/j.molcel.2018.07.036. 30197300 PMC6230553

[acel70257-bib-0022] Kim, M. , Y. Qie , J. Park , and C. H. Kim . 2016. “Gut Microbial Metabolites Fuel Host Antibody Responses.” Cell Host & Microbe 20: 202–214. 10.1016/j.chom.2016.07.001. 27476413 PMC4982788

[acel70257-bib-0023] Levy, M. , E. Blacher , and E. Elinav . 2017. “Microbiome, Metabolites and Host Immunity.” Current Opinion in Microbiology 25: 8–15. 10.1016/j.mib.2016.10.003. 27883933

[acel70257-bib-0024] Martinez‐Zamundio, R. I. , H. K. Dewald , T. Vasilopoulos , et al. 2021. “Senescence‐Associated β‐Galactosidase Reveals the Abundance of Senescent CD8+ T Cells in Aging Humans.” Ageing Cell 20: e13344. 10.1111/acel.13344. PMC8135084 33939265

[acel70257-bib-0025] Monaghan, T. M. , N. A. Duggal , E. Rosati , et al. 2021. “A Multi‐Factorial Observational Study on Sequential Fecal Microbiota Transplant in Patients With Medically Refractory Clostridioides Difficile Infection.” Cells 10: 3234. 10.3390/cells10113234. 34831456 PMC8624539

[acel70257-bib-0047] Ni, Y. F. , J. Wang , X. L. Yan , et al. 2010. “Histone Deacetylase Inhibitor, Butyrate, Attenuates Lipopolysaccharide‐Induced Acute Lung Injury in Mice.” Respiratory Research 11: 33. 10.1186/1465-9921-11-33. 20302656 PMC2848144

[acel70257-bib-0027] Pollizzi, K. N. , C. H. Patel , H. I. Sun , et al. 2015. “M TORC1 and m TORC2 Selectively Regulate CD8 T Cell Differentiation.” Journal of Clinical Investigation 125: 2090–2108. 10.1172/JCI77746. 25893604 PMC4463194

[acel70257-bib-0028] Raza, K. , A. Sharma‐Oates , L. Padyukov , et al. 2025. “Specific Features of Immune Ageing Are Detected in the Earliest Stages in Rheumatoid Arthritis Development.” eBioMedicine 119: 105900.40908179 10.1016/j.ebiom.2025.105900

[acel70257-bib-0029] Rivière, A. , M. Selak , D. Lantin , F. Leroy , and L. De Vuyst . 2016. “Bifidobacteria and Butyrate‐Producing Colon Bacteria: Importance and Strategies for Their Stimulation in the Human Gut.” Frontiers in Microbiology 7: 979. 10.3389/fmicb.2016.00979. 27446020 PMC4923077

[acel70257-bib-0030] Rosser, E. C. , C. J. M. Piper , D. E. Matei , et al. 2020. “Microbiota‐Derived Metabolites Supress Arthritis by Amplifying Aryl‐Hydrocarbon Receptor Activation in Regulatory B Cells.” Cell Metabolism 31: 837–851. 10.1016/j.cmet.2020.03.003. 32213346 PMC7156916

[acel70257-bib-0031] Salazar, N. , S. Arboleya , T. Navarro , et al. 2019. “Age‐Associated Changes in Gut Microbiota and Dietary Components Related With the Immune System in Adulthood and Old Age: A Cross Sectional Study.” Nutrients 11: 17. 10.3390/nu11081765. PMC6722604 31370376

[acel70257-bib-0032] Saul, D. , R. L. Kosinsky , E. J. Atkinson , et al. 2022. “A New Gene Set Identifies Senescent Cells and Predicts Senescence‐Associated Pathways Across Tissues.” Nature Communications 13: 4827. 10.1038/s41467-022-32552-1. PMC9381717 35974106

[acel70257-bib-0033] Sayegh, S. , C. H. Fantecelle , P. Laphanuwat , et al. 2024. “Vitamin D3 Inhibits p38 MAPK and Senescence‐Associated Inflammatory Mediator Secretion by Senescent Fibroblasts That Impacts Immune Responses During Ageing.” Aging Cell 23: e14093. 10.1111/acel.14093. 38287646 PMC11019144

[acel70257-bib-0044] Scheppach, W. , H. Sommer , T. Kirchner , et al. 1992. “Effect of Butyrate Enemas on the Colonic Mucosa in Distal Ulcerative Colitis.” Gastroenterology 103: 51–56. 10.1016/0016­5085(92)91094-k. 1612357

[acel70257-bib-0034] Sun, Q. , X. Chen , J. Ma , et al. 2011. “Mammalian Target of Rapamycin Up‐Regulation of Pyruvate Kinase Isoenzyme Type M2 Is Critical for Aerobic Glycolysis and Tumorgrowth.” Proceedings of the National Academy of Sciences of the United States of America 108: 4129–4134. 10.1073/pnas.1014769108. 21325052 PMC3054028

[acel70257-bib-0035] Tchkonia, T. , Y. Zhu , J. van Deursen , J. Campisi , and J. L. Kirkland . 2013. “Cellular Senescence and the Senescent Secretory Phenotype: Therapeutic Opportunities.” Journal of Clinical Investigation 123: 966–972. 10.1172/jci64098. 23454759 PMC3582125

[acel70257-bib-0036] Thevaranjan, N. , A. Puchta , C. Schulz , et al. 2017. “Age‐Associated Microbial Dysbiosis Promotes Intestinal Permeability, Systemic Inflammation, and Macrophage Dysfunction.” Cell Host & Microbe 21: 455–466. 10.1016/j.chom.2017.03.002. 28407483 PMC5392495

[acel70257-bib-0037] Usami, M. , K. Kishimoto , A. Ohata , et al. 2008. “Butyrate and Trichostatin A Attenuate Nuclear Factor kappaB Activation and Tumor Necrosis Factor Alpha Secretion and Increase Prostaglandin E2 Secretion in Human Peripheral Blood Mononuclear Cells.” Nutrition Research 28: 321–328. 10.1016/j.nutres.2008.02.012. 19083427

[acel70257-bib-0043] Van der Beek, C. M. , J. G. Bloemen , M. A. Van den Broek , et al. 2015. “Hepatic Uptake of Rectally Administered Butyrate Prevents an Increase in Systemic Butyrate Concentrations in Humans.” Journal of Nutrition 145: 2019–2024. 10.3945/jn.115.211193. 26156796

[acel70257-bib-0039] Xiao, F. H. , Q. Yu , Z. L. Deng , et al. 2022. “ETS1 Acts as a Regulator of Healthy Aging via Decreasing Ribosomal Activity.” Science Advances 8: eabf2017. 10.1126/sciadv.abf2017. 35476452 PMC9045719

[acel70257-bib-0040] Youn, J. , M. K. Jung , H. T. Yu , et al. 2019. “Increased Frequency of CD4^+^CD57^+^ Senescent T Cells With Newly Diagnosed Acute Heart Failure: Exploring New Pathogenic Mechanisms With Clinical Relevance.” Scientific Reports 9: 12887. 10.1038/s41598-019-49332-5. 31501486 PMC6733929

[acel70257-bib-0046] Zhang, L. T. , Y. M. Yao , J. Q. Lu , et al. 2007. “Sodium Butyrate Prevents Lethality of Severe Sepsis in Rats.” Shock 27: 672–677. 10.1097/shk.0b013e31802e3f4c. 17505308

[acel70257-bib-0041] Zhang, J. , H. Tianhui , L. Xue , et al. 2021. “Senescent T Cells: A Potential Biomarker and Target for Cancer Therapy.” eBioMedicine 68: 103409. 10.1016/j.ebiom.2021.103409. 34049248 PMC8170103

[acel70257-bib-0042] Zukowski, E. , M. Sannella , J. D. Rockhold , et al. 2023. “STAT3 Modulates CD4^+^ T Mitochondrial Dynamics and Function in Aging.” Aging Cell 22: e13996. 10.1111/acel.13996. 37837188 PMC10652300

