# Supplementary material for: Defining Microbiota‐Derived Metabolite Butyrate as a Senomorphic: Therapeutic Potential in the Age‐Related T Cell Senescence
Source: Aging Cell. 2025 Nov 7;24(12):e70257. doi: 10.1111/acel.70257 (PMC12686592; doi:10.1111/acel.70257)
Supplement: Supplementary file 1 — Table S1: T cell subset distribution post 3 day cell culture with Butyrate. Figure S1: A cellular model of proliferation‐induced T cell senescence. Figure S2: Regulation of senescence phenotype by butyrate in aged CD4 T cells. Figure S3: Effect of butyrate on nuclear factor kappa B (NFκB), p‐38 MAPK and m TOR signalling in aged CD4 T cells. Figure S4: Effect of butyrate on mitochondrial mass and ROS production in CD4 T cells. [file ACEL-24-e70257-s001.pdf]

	Control	Butyrate	P value
CD4 T cells (%)	57.77 (±17.07)	62.24 (±14.82)	0.49
Naïve CD4 T cells (%)	47.82 (±14.55)	44.40 (± 16.33)	0.81
CM CD4 T cells (%)	24.08 (±12.38)	26.32 (±18.21)	0.87
EM CD4 T cells (%)	20.64 (±16.51)	21.92 (±20.44)	0.96
EMRA CD4 T cells (%)	8.25 (±11.49)	6.97 (±13.94)	0.66
$CD57^{+ve}$ CD4 T cells (%)	4.23 (±2.07)	3.35 (±2.17)	0.34
$Ki67+^{ve}$ CD4 T cells (%)	5.68 (± 3.11)	4.21 (± 1.89)	0.53
CD8 T cells (%)	38.74 (±16.16)	38.90 (±13.70)	0.79
Naïve CD8 T cells (%)	26.03 (±13.39)	27.39 (±12.34)	0.62
CM CD8 T cells (%)	19.24 (±17.35)	19.91 (±22.47)	0.97
EM CD8 T cells (%)	29.44 (±23.64)	31.37 (±11.28)	0.58
EMRA CD8 T cells (%)	11.09 (±8.23)	10.21 (±11.9)	0.90
$CD57^{+ve}$ CD8 T cells (%)	3.50 (±1.89)	4.34 (1.97)	0.71
$Ki67+^{ve}$ CD8 T cells (%)	4.8 (± 2.76)	4.21 (± 1.54)	0.68

Supplementary Table 1 . T cell subset distribution post 3 day cell culture with Butyrate

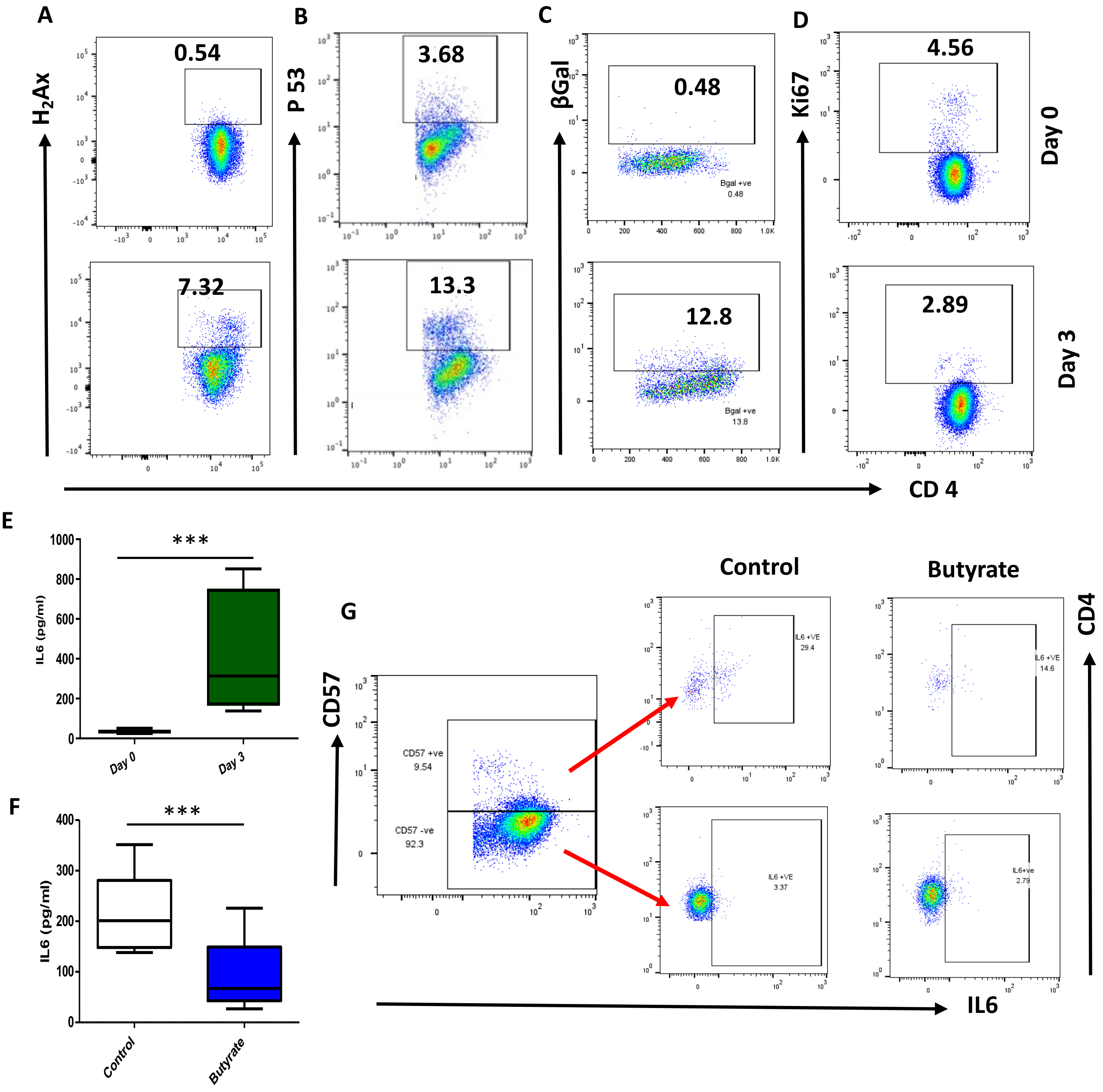

Supplementary Figure 1. A cellular model of proliferation-induced T cell senescence.

In-vitro culture of T cells in CD3-coated wells for 3 days representative flow cytometry images for Day 0 vs Day 3 (A) DNA damage (H2Ax) marker (B) replicative arrest mediated by upregulation of tumor-suppressor pathway p53. (C) Spider βGal expressing cells (D) Ki67 expressing cells. (E) Senescent cells secrete products (SASP) that are important contributors to aging and an upregulation of SASP factor IL-6 has been observed in cell-culture supernatant at day3 indicative of three independent experiments. (F) The secretion of IL6 from CD3-treated young T cells from 6 healthy young participants in the presence of 1 M butyrate. (G) Gating strategy for IL6 production by CD57^{+ve} senescent and CD57^{-ve} non senescent CD3-treated CD4 T cells in the presence of 1M butyrate. Statistical analysis was performed by two-tailed paired student's t-test $*P < 0.05$, $**p < 0.01$, $***p < 0.001$.

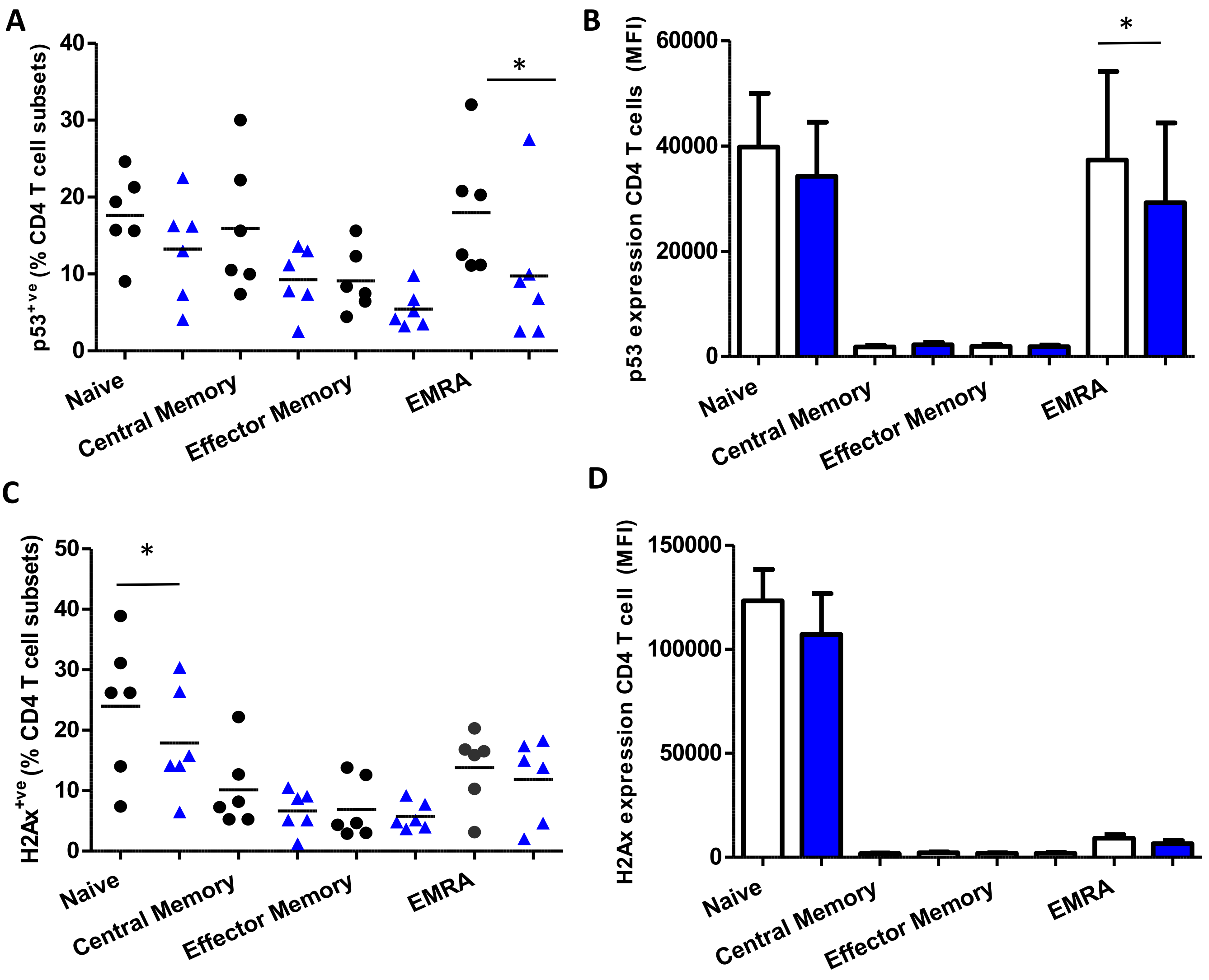

Supplementary Figure 2. Regulation of senescence phenotype by butyrate in aged CD 4 T cells

In-vitro culture of PBMCs in CD3-coated wells for 3 days in the presence/absence of SCFA butyrate for (A) frequency of phosphorylated p53 expressing CD4 T cell subsets (B) p 53 expression levels in CD 4 T cell subsets (C) frequency of γH2AX expressing CD4 T cell subsets (D) γH2AX expression levels (MFI) in CD4 T cell subsets. A two-tailed paired Student's t-test was used to perform the statistical analysis. Data are shown as the mean ± SD ***$p < 0.001$. n =6 healthy old donors per experiment.

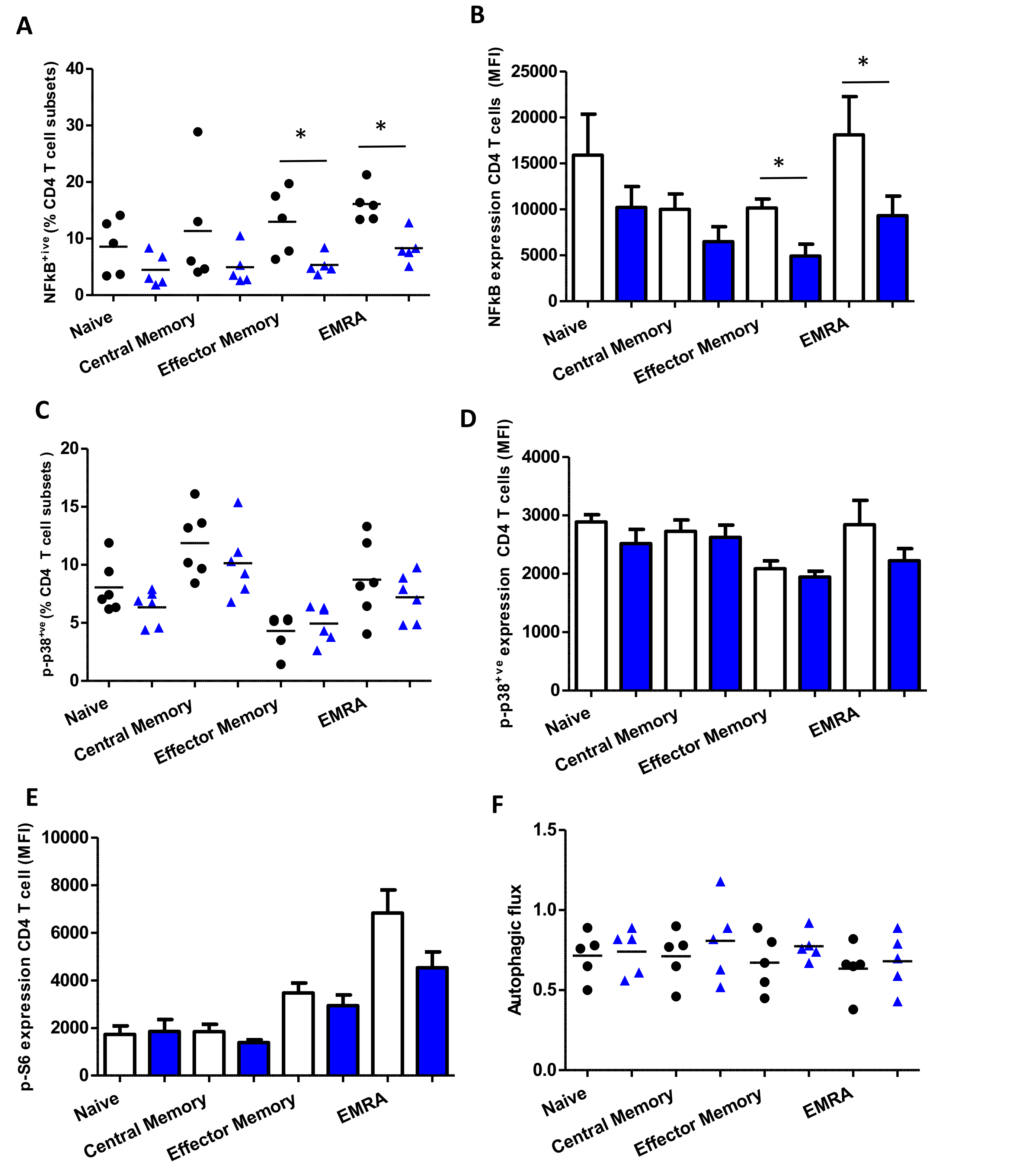

Supplementary Figure 3. Effect of butyrate on nuclear factor kappa B (NFκB), p-38 MAPK and m TOR signalling in aged CD4 T cells. In-vitro culture of T cells in CD3-coated wells for 3 days in the presence/absence of SCFA butyrate for (A) frequency of phosphorylated NF-kB expressing CD4 T cell subsets. (B) NF-kB expression levels in CD4 T cell subsets (C) frequency of phosphorylated p38 expressing CD4 T cell subsets. (D) p-p38 expression levels in CD4 T cell subsets (E) p S6 mean fluorescence intensity (MFI) in CD4 T cell subsets (naïve, central memory, effector memory, EMRA). (F) Autophagic flux in CD4 T cell subsets (naïve, central memory, effector memory, EMRA). A two-tailed paired Student's t-test was used to perform the statistical analysis. Data are shown as the mean ± SD $^{*}p = 0.05$, $^{**}p < 0.01$. n =6 healthy old donors per experiment.

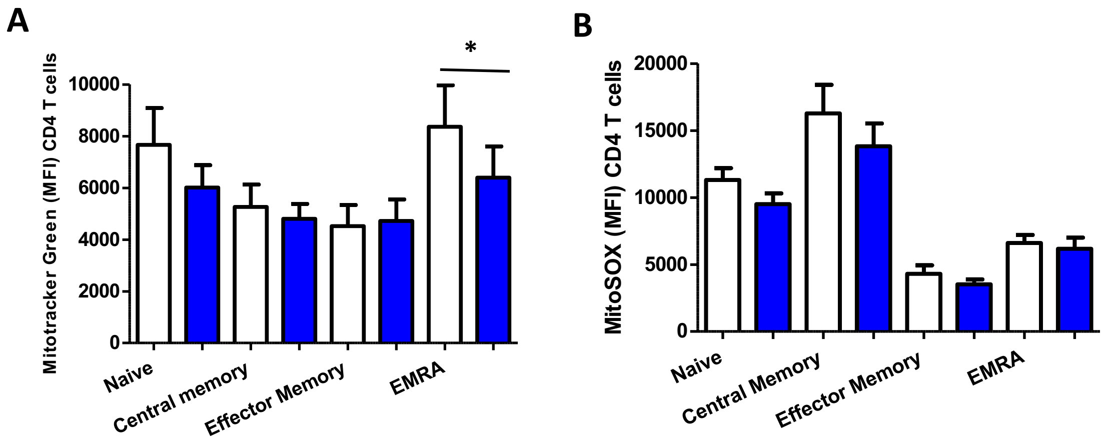

Supplementary Figure 4. Effect of butyrate on mitochondrial mass and ROS production in CD4 T cells.

In vitro culture of T cells in CD3-coated wells for 3 days in the presence/absence of SCFA butyrate and stimulation. **(A)** MitoTracker™ green expression levels in aged CD4 T cell subsets **(B)** MitoSOX™ red expression levels in aged CD4 T cell subsets. Statistical analysis was performed by a two-tailed paired Student's t-test. Data are shown as the mean ± SD *p <0.05, **p < 0.01.
